# Stereotactic Body Radiotherapy for Metachronous Multisite Oligo-Recurrence: A Long-Surviving Case with Sequential Oligo-Recurrence in Four Different Organs Treated Using Locally Radical Radiotherapy and a Review of the Literature

**DOI:** 10.1155/2012/713073

**Published:** 2012-10-23

**Authors:** Hiroshi Onishi, Masatoki Ozaki, Kengo Kuriyama, Takafumi Komiyama, Kan Marino, Masayuki Araya, Ryo Saito, Shinichi Aoki, Yoshiyasu Maehata, Licht Tominaga, Mitsuhiko Oguri, Iori Watanabe, Kojiro Onohara, Meguru Watanabe, Naoki Sano, Tsutomu Araki

**Affiliations:** ^1^Department of Radiology, School of Medicine, Yamanashi University, 1110 Shimokato, Chuo City, Yamanashi 409-3898, Japan; ^2^Department of Radiology, Shizuoka Municipal Shimizu Hospital, Shizuoka 424-8636, Japan

## Abstract

Stereotactic body radiotherapy (SBRT) for oligometastases represents a recent trend in radiation oncology. While abundant data are available regarding the use of SBRT for the treatment of lung or liver oligometastases from various retrospective series and prospective trials, relatively little information has been accumulated for the treatment of oligometastases at sites other than the lungs and liver, particularly for sequential oligometastases in multiple organs. Oligometastases with primary lesions controlled is called “oligo-recurrence.” We describe herein the case of a lung cancer patient who developed repeated oligo-recurrence at multiple sites that were each controlled by radical radiotherapy and achieved long-term survival and discuss the merits of locally aggressive radiotherapy for this type of disease condition with reviewing the literature. Although further investigation should be undertaken to clarify the benefits, objectives, and methods of SBRT for the treatment of oligometastases, we believe utilization of SBRT may be worthwhile for patients with remote metastases who hope for treatment to acquire better local control and possible longer survival.

## 1. Introduction

Interest has been increasing in the use of local therapy for metastases in recent years, likely due to improvements in systemic therapy [1–5]. In a selected population of oligometastatic patients, surgical metastasectomy may prolong survival and data in the literature support this observation. Survival benefits were being reported for complete resection of metastatic lung tumors even in the 1990s. The International Registry of Lung Metastases (IRLM) reported that 5-year overall survival for patients with complete resection of metastatic lung tumors was 36%, compared with 13% for patients without, suggesting complete removal or ablation of metastatic lesions as an important predictor of long-term survival [2].

Although surgical metastasectomy remains the most common of the local therapies, representing the first-line standard, nonsurgical alternatives such as thermal ablation and stereotactic body radiotherapy (SBRT) have become increasingly popular as options for patients who are not surgical candidates or who decline surgery. This is because these options are generally less invasive than surgery and have demonstrated considerable promise in eradicating macroscopic tumor. The main aim of SBRT is to acquire better local control of the tumor by providing a higher dose of irradiation to a specified area during a short period. SBRT was initially developed in Sweden and Japan [6, 7]. SBRT has been available for more than 10 years and is gaining clinical interest as a means of achieving local radical treatment of tumors in various organs, particularly for patients with stage I non-small-cell lung cancer (NSCLC) [8–13], not only in medically inoperable patients, but also in operable patients [14].

SBRT for oligometastases represents a recent trend in radiation oncology [15–17]. Concerning the survival benefit of locally aggressive radiotherapy for oligometastases the largest experience has been accumulated for patients with brain metastases treated by stereotactic radiosurgery. In 2005, the American Society for Therapeutic Radiology and Oncology (ASTRO) systematically reviewed the evidence for the use of stereotactic radiosurgery in adult patients with brain metastases, and concluded that radiosurgery boost with whole-brain radiotherapy improved survival in patients with a single brain metastasis [18]. Niibe et al. also indicated that patients with oligometastases and no extrathoracic lesions could receive survival benefits from SBRT [19].

Milano et al. analyzed a subset of 121 patients treated with curative-intent SBRT for a limited number of extracranial metastases [16]. The results of their study showed that patients fared well with respect to survival and disease control with aggressive SBRT, even after local failure and/or the development of new metastases. While abundant data are available regarding the use of SBRT for the treatment of lung or liver oligometastases from various retrospective series [20, 21] and prospective trials [16, 22, 23], relatively little information has been accumulated for the treatment of oligometastases at sites other than the lungs and liver, particularly for sequential oligometastases in multiple organs.

Oligometastases with primary lesions controlled is called “oligo-recurrence”, that was first noted by Niibe et al. [24, 25]. We describe herein the case of a lung cancer patient who developed repeated oligo-recurrence at multiple sites that were each controlled by radical radiotherapy and achieved long-term survival, and discuss the merits of locally aggressive radiotherapy for this type of disease condition.

## 2. Clinical Case

Although SBRT in the strict definition generally includes large fraction size (generally not less than 5 Gy) and a short treatment-duration (generally within 3 weeks), we call the radiotherapy for the adrenal or abdominal lymph node metastases, that was done in a stereotactic manner but with 3 Gy in every fractions during over 3 weeks, “SBRT” in this case report.

In October 2006, a 68-year-old Japanese man presented with T2N2M0 adenosquamous carcinoma in the right upper lobe of the lung. The patient underwent complete tumor resection with right upper lobectomy and mediastinal lymph nodes dissection. He had received adjuvant chemotherapy (four cycles of carboplatin; area under the curve (AUC) = 5 (1000 mg/body) on day 1 of a 21-day cycle) with weekly paclitaxel (1000 mg/m^2^).

**Figure 1:**
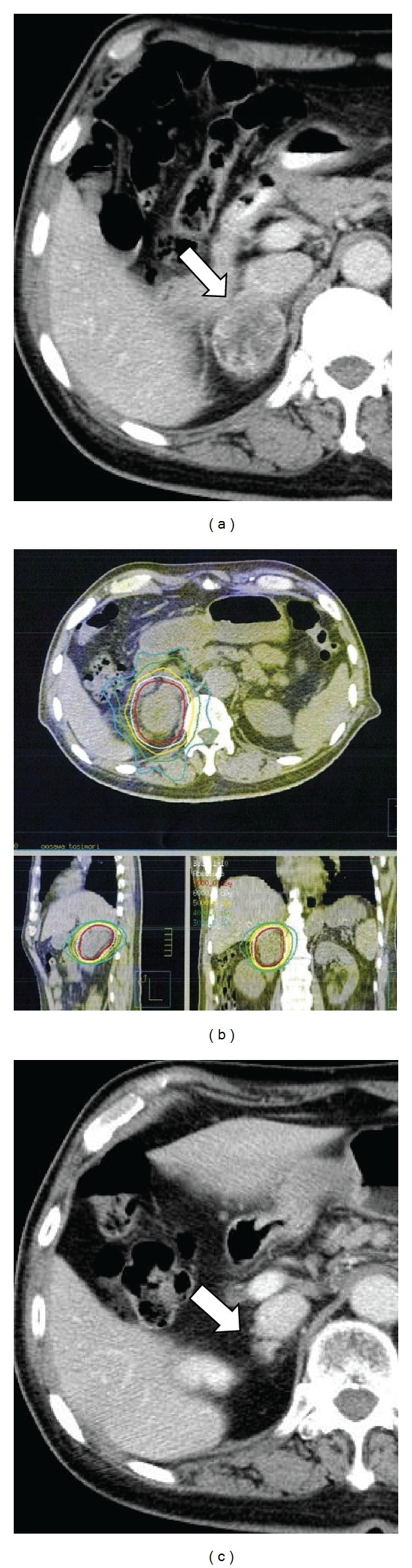
(a) CT of right adrenal metastasis (arrow) before SBRT. (b) Dose distribution made with 10 noncoplanar beams for SBRT. Isodose lines show total doses (in 25 fraction) of 70 Gy, 60 Gy, 50 Gy, and 40 Gy, in 10 fractions, respectively, from the innermost area. The 30-Gy isodose line overlapped at the second portion of the duodenum with the 40-Gy isodose line of the SBRT for right adrenal metastasis, resulting in grade 2 duodenitis 1 month after SBRT. (c) CT at 6 months after SBRT, showing partial response of the lesion (arrow).

**Figure 2:**
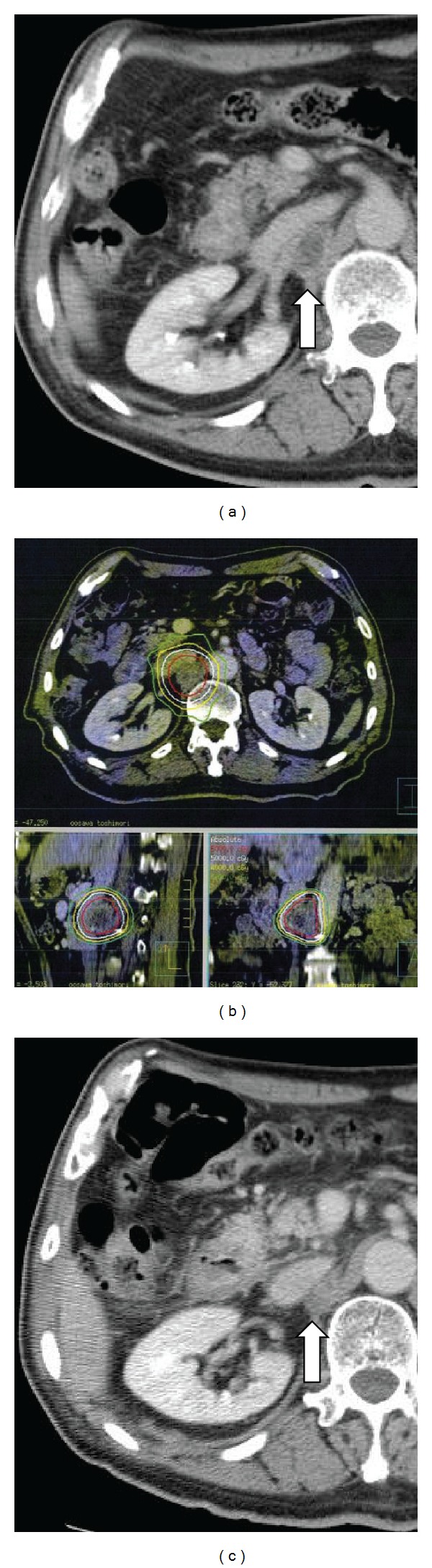
(a) CT of right para-aortic lymph node metastasis (arrow) before SBRT. (b) Dose distribution made with 10 noncoplanar beams for SBRT. Isodose lines show total doses (in 20 fractions) of 60 Gy, 50 Gy, 40 Gy, and 30 Gy, respectively, from the innermost area. (c) CT at 4 months after SBRT, showing partial response of the lesion (arrow).

**Figure 3:**
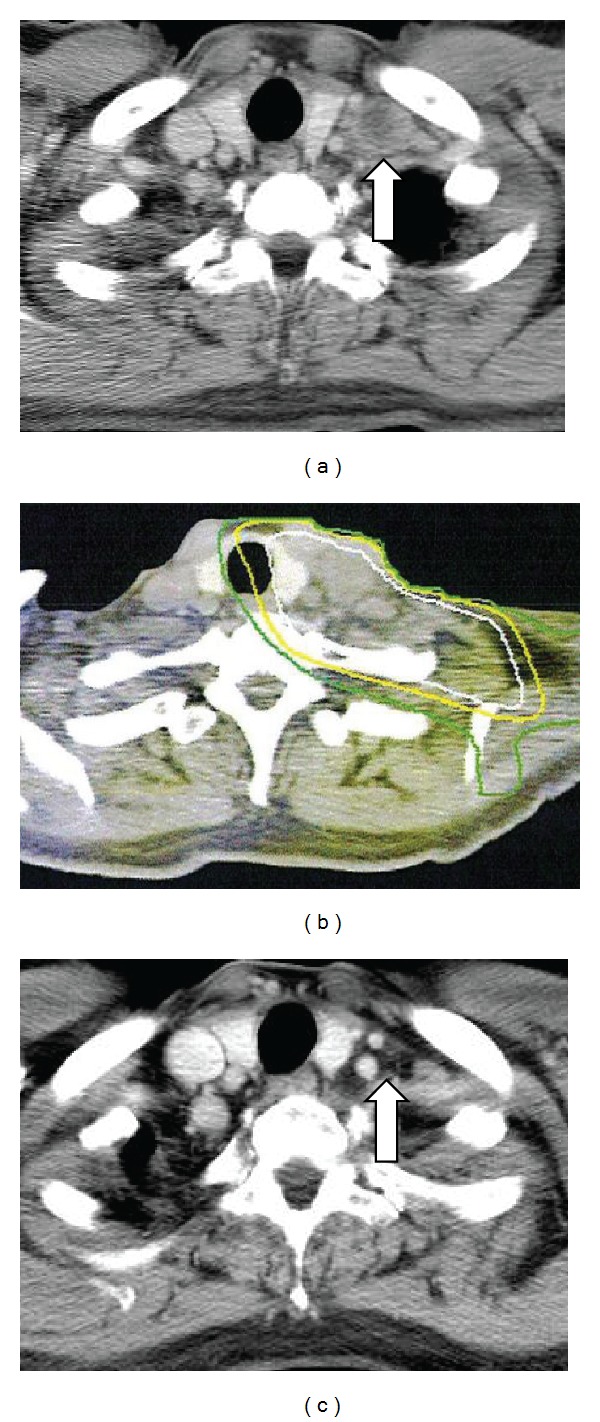
(a) CT of left supraclavicular lymph node metastases (arrow) before SBRT. (b) Dose distribution made with 4 coplanar beams for conventional radiotherapy. Isodose lines shows total doses (in 29 fracions) of 50 Gy, 40 Gy, and 30 Gy, respectively, from innermost area. (c) CT at 1 months after the RT, showing complete response of the lesion (arrow).

**Figure 4:**
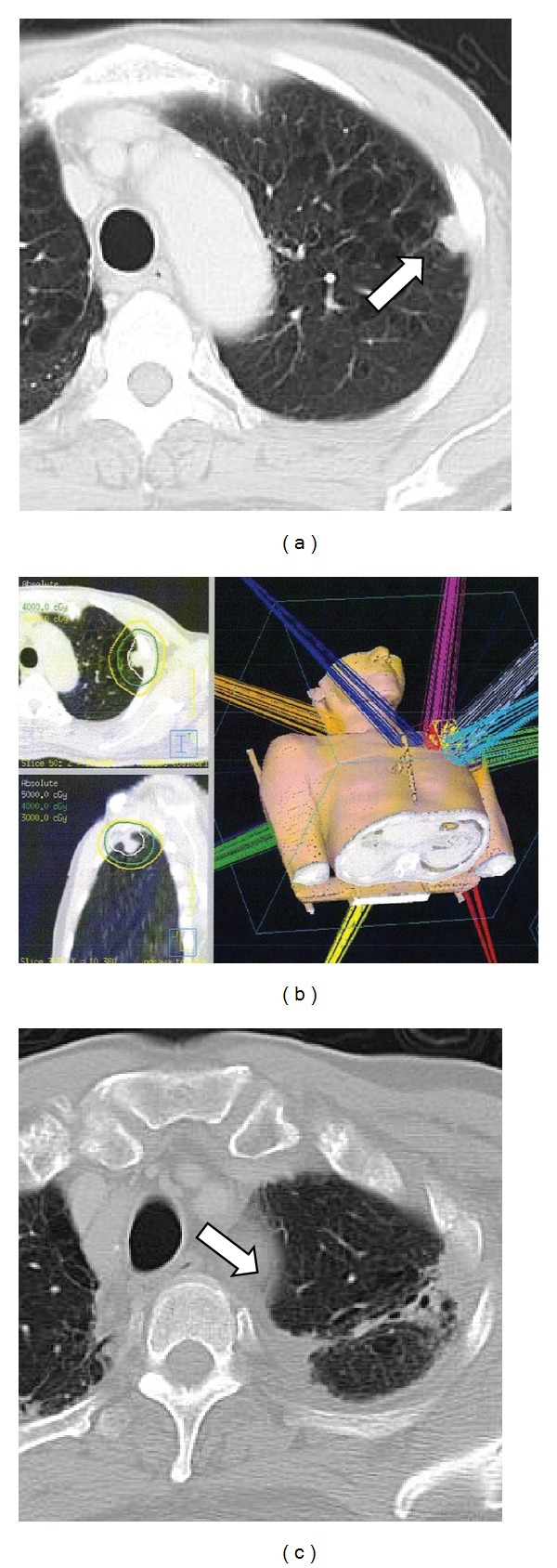
(a) CT of the left lung metastasis (arrow) before SBRT. (b) Dose distribution made with 10 noncoplanar beams for SBRT. The isodose lines shows total doses (in 4 fractions) of 50 Gy, 40 Gy, and 30 Gy, respectively, from innermost area. (c) CT at 2 years after SBRT, showing partial response of the lesion (arrow) with focal radiation fibrosis.

A right adrenal mass was found on routine computed tomography (CT) in March 2007 and was diagnosed as a solitary right adrenal metastasis by ^18^F-fluoro-2 deoxy-D-glucose (FDG)-positron emission tomography (PET). Although the patient had taken tegafur-uracil (UFT) at 4.0 g/day for three months, the metastasis continued to enlarge (40 mm in diameter by June 2007; Figure 1(a)). The patient was therefore referred to our clinic for SBRT. Although other regimens of chemotherapy were considered, the patient wished to undergo SBRT as local intensive therapy. He showed a very positive and cheerful demeanor. During the SBRT planning sessions, the patient was trained in voluntary breath-holding during the inspiration phase using a respiratory indicator [26] to minimize the adrenal respiratory motions during irradiation [27]. Planning target volume (PTV) was determined as the gross tumor volume (GTV) of the right adrenal mass plus the personal internal margin, with an additional margin of 2 mm to compensate for intrasession reproducibility and to provide a safety margin. Precise reproducibility of tumor position in this patient under voluntary breath-holding was measured on repeated CT. Tumor position was adjusted to the planned position before every session using the CT on rails taken in the vicinity of the tumor. Ten different noncoplanar static beams were used for irradiation. The radiation port was made with dynamic sliding multileaves adjusted with 3 mm margins around the border of the PTV. Dose constraints of normal tissue were defined for the intestine and spinal cord. For the intestine, volumes with dose >52.5 Gy and >43.2 Gy in 10 fractions (biologically effective dose (BED) = 144.4 Gy and 105.0 Gy, resp., *α*/*β* = 3 Gy) were restricted within 10 mL and 100 mL, respectively. For the spinal cord, maximum dose was restricted to <36 Gy in 10 fractions (BED = 79.2 Gy, *α*/*β* = 3 Gy). These criteria represent a modification of the dose constraints provided in the protocol of the Japanese Clinical Oncology Group (JCOG)-0403 study, a prospective study of SBRT for stage I NSCLC. A total dose to the isocenter of 75 Gy in 25 fractions over 36 days was delivered using a 10-MV X-ray from June to July 2007. Isodose lines on CT are shown in Figure 1(b). The reason for the middle fraction size (3 Gy) was to avoid serious toxicity affecting the duodenum, because the second portion was included in the high-dose area. Administration of UFT was stopped before the start of the SBRT. After completion of SBRT, daily oral administration of tegafur-gimeracil-oteracil potassium (TS-1) was initiated at 80 mg/body. The patient complained of mild epigastralgia in December 2007, and grade 1 duodenitis was observed under fiberscopy. Symptoms improved with administration of oral antacids. In February 2008, the right adrenal tumor had decreased in size sufficiently to meet the criteria for partial response (PR; Figure 1(c)), but right para-aortic lymph node swelling (diameter, 30 mm; Figure 2(a)) was found on CT. This lesion was considered to represent a new metastasis of lung cancer. At this point of time, we informed the patient that he had systemic multiple metastases and that complete cure might be difficult. However, he was elected to undergo further local treatment and a second course of SBRT was therefore performed for this new lesion. The method of the SBRT was similar to that for the right adrenal metastasis. A total dose to the isocenter of 60 Gy shown in Figure 2(b) in 20 fractions over 28 days was delivered. A small overlap of treated volumes was produced between the first and second courses of SBRT, affecting the second portion of the duodenum, but dose constraints were not exceeded. No toxicities in relation to the second course of SBRT were identified. Administration of TS-1 was stopped before the start of second SBRT and resumed after completion. In July 2008, the patient complained of acute hoarseness, and CT showed a new lymph node swelling in the left supraclavicular fossa (diameter, 25 mm; Figure 3(a)) and a left upper lung nodule (diameter, 20 mm; Figure 4(a)), although the right para-aortic lymph node lesion had decreased in size to represent PR (Figure 2(c)). Aspiration cytology was performed from the left supraclavicular fossa, revealing adenosquamous carcinoma cells. We considered that the condition of the patient at this time represented a more difficult stage and that the potential merits of local treatment were likely to be reduced. However, the patient again insisted on local radical treatment and we were persuaded by his eagerness. We first tried to control the left supraclavicular lesion. A total dose to the isocenter of 52.2 Gy in 29 fractions (shown in Figure 3(b); 1.8 Gy/fraction, twice a day, accelerated hyperfractionation) over 22 days was delivered to only the swollen left supraclavicular lymph node using conventional radiotherapy techniques. The reason why we did not use SBRT for the lesion was to avoid an adverse effect on the brachial plexus. Administration of TS-1 was continued during and after the sessions until February 2009. Hoarseness improved and FDG-PET-CT studies 1 month after this third course of radiotherapy showed marked reductions in size of the left supraclavicular lesion (Figure 3(c)) with no accumulation of FDG and no other abnormal accumulations. SBRT for left upper lobe metastases was then performed in September 2008. SBRT for the left upper lung lesion was performed using a similar method to the previous right adrenal and para-aortic lesions, but the prescribed dose was 48 Gy in four fractions over 4 days to cover 95% of the PTV (Figure 4(b)). The tumor decreased in size to PR (Figure 4(c)) and has not progressed since. No other metastases have been identified since the completion of these four sessions of radiotherapy, including 3 courses of SBRT. Although fracture of the left rib within the PTV of the SBRT for the left lung metastases and idiopathic right pneumothorax occurred in March 2011 and August 2011, respectively, the patient has remained very well without cancer recurrence and has enjoyed hobby (dancing) cheerfully as recently as June 2012.

## 3. Discussion

Recent evidence suggests the presence of an oligometastatic state, where metastases are limited in both number and site. Weichselbaum and Hellman first proposed this concept of oligometastases as a state of “restricted tumor metastatic capacity” in 1995 [28], ushering in a paradigm shift in the strategy of cancer treatment.

Oligometastases has been hypothesized to represent a state of distant metastases in which local therapies, such as resection or radiation, may offer cure in some patients [29–31]. Locally curative treatment of oligometastases is regarded as an important resource for improving survival in a clinically significant subset of cancer patients [32, 33]. Local control of oligometastatic lesions may also slow or prevent further metastatic progression [34].

The maximum number of lesions that can be present to meet the definition of oligometastases has not been officially defined, but the number and organs affected by tumors is generally defined as ≤5 lesions in ≤2 organs. Salama et al. undertook a prospective study of SBRT for patients with metastases in 1–5 sites and reported 2-year progression-free and overall survival rates of 22.0% and 56.7%, respectively [22]. They concluded that patients with 1–5 metastases can be safely treated at multiple body sites and may benefit from SBRT. Aggressive treatment of such oligometastatic lesions can often be considered curative, because this treatment has been seen to prolong disease-free survival.

Several institutions have been actively using hypofractionated SBRT as a less-invasive locally curative treatment for oligometastases [32, 35, 36]. SBRT is mostly practiced for primary stage I NSCLC in Japan, followed by metastatic lung cancer, then metastatic liver cancer [37].

We will now provide an overview and discussion of SBRT for oligometastases in relation to the present case with adrenal, lymph node, and lung metastases.

Concerning SBRT for lung metastases, main reported outcomes are summarized in Table 1. The number of lung metastases of the enrolled patients distributed from 1 to 3 in most of the reports. Multiple retrospective [1, 5, 15, 20, 38, 39] and prospective [40–46] studies have shown promising local control (LC) with SBRT, with some investigations reporting LC rates of approximately 90%. Most studies have observed very low rates of serious toxicities. Norihisa et al. [38] reported that 43 metastatic lung tumors in 34 patients achieved a 2-year local control rate of 90% and a 2-year overall survival rate of 84.3% as a result of SBRT at 48–60 Gy in 4-5 fractions to the isocenter. Le et al. recently reported the results of a phase II trial using SBRT to a dose of 50 Gy in 10 fractions in the treatment of oligometastatic disease [41]. Lung metastases were treated in 41% and thoracic lymph nodes in 20% of patients. The 2-year local control rate for all treated lesions was 67%. Similarly, investigators from Heidelberg treated 61 patients with 71 lung metastases using single-fraction SBRT to an isocenter dose of 12–30 Gy and reported an actuarial local control rate of 74% at 2 years [43]. Hoyer et al. completed a phase II trial of SBRT to a dose of 45 Gy in 3 fractions for treatment of colorectal metastases, primarily involving the lung and liver. The actuarial 2-year local control rate in that series was 86% [44]. Rusthoven et al. reported a phase I/II prospective study of SBRT for metastatic lung tumors. Thirty-eight patients with 63 lesions treated with SBRT achieved a 2-year local control rate of 96%, but a 2-year overall survival of only 39% [46]. One of the important reasons behind this poor prognosis with SBRT though the good local control similar to rates reported using 60–66 Gy in 3 fractions for primary NSCLC [47] might be that the prospective study included patients with extrapulmonary lesions. McCammon et al. also reported excellent local control rates with a nominal dose of ≥54 Gy and suggested a dose-control relationship within the range of SBRT doses applied [48]. These results suggest that the higher, more intense dose of SBRT used in the current series likely contributed to the higher rate of local control rate observed, although patient selection bias is always a potential confounder in comparisons across studies.

In contrast to SBRT for most lung or liver metastases, careful attention must be paid to the dose and fractions for areas of intestine surrounding the tumor such as the present case. In the presented case, although we referred to the dose constraints provided in the protocol of the JCOG-0403 study and fortunately the patient had not suffer severe bowel toxicity, the dose constraint for intestines may be rather high from a viewpoint of conventional radiotherapy because the intestine is a serial organ, volume effect would not be large, and the maximum dose or near maximum dose would be the major concern. The author have experienced a serious gastric ulcer event occurring after SBRT (60 Gy in 10 fractions) delivered with concomitant vinorelbine in a patient with left adrenal metastasis of lung cancer [49]. The true dose constraint for intestines in the hypofractionated radiotherapy should be more investigated hereafter. Recently, the benefits of dose concentration by Cyberknife to avoid normal tissues receiving high doses have been reported in SBRT for tumors located close to the bowel or esophagus [50–54].

Concerning adrenal metastases, they are increasingly being detected incidentally during followup or at the time of initial presentation with continuing progress in imaging techniques. A relevant meta-analysis reported improved survival after adrenalectomy in patients affected by adrenal metastases from lung cancer, achieving durable long-term survival in approximately 25% of cases [55]. Although SBRT is commonly accepted as a safe and fairly effective treatment for controlling small cancer lesions, SBRT for the adrenal gland has been described in only a few studies summarized in Table 2 [56–59]. Chawla et al. [56] and Casamassima et al. [57] showed that adrenal SBRT may be considered a radical therapy not influenced by parameters such as primary tumor, synchronous or metachronous status, uni- or bilateral lesions, oligometastatic disease, or target volume. Oshiro et al. suggested that radiotherapy may contribute to the survival of patients with adrenal metastasis from lung cancer [58]. Milano et al. analyzed a subset of 121 patients treated with curative-intent SBRT for limited metastases and emphasized the advantages of SBRT versus surgery for the treatment of adrenal metastases, such as low incidence of side effects, good tolerability, and the noninvasive nature of treatment, allowing application in elderly or medically inoperable patients [60]. Although surgery resulted in appreciably better survival, this might, in part, have resulted from patient selection, such as patients with less bulky adrenal metastases and/or without additional metastases to other organs. The poor outcomes of patients with adrenal metastases treated using curative-intent SBRT compared with outcomes for patients without adrenal metastases [43] suggest that perhaps metastases to the adrenal glands are associated with a greater risk of occult metastatic disease, and such patients are thus less likely to benefit from curative-intent therapy.

Concerning SBRT for oligometastases to lymph nodes, conventional fractionated nonstereotactic radiotherapy is generally believed to attain poorer results, because doses are limited by normal tissue tolerance. Although several articles have dealt with conventional radiotherapy for isolated para-aortic lymph node recurrences from cervical cancer, most have reported only survival rates [25, 61–63]. Progressive disease after conventional radiotherapy in the para-aortic lymph node-treated area was reported to be 33, 50% in two studies [64, 65]. Whereas most patients with metastases to abdominal nodes are unfit for surgery, SBRT is known to lead to high local control rates up to 90% [32], which may in turn allow increased survival and better quality of life. SBRT for metastases to abdominal lymph nodes has rarely been reported, with only a few articles reporting on this as a specific topic [50–52] summarized in Table 3 and with most only including a few cases in a mixed series [45, 53, 54, 66, 67]. One of the reasons why SBRT or any form of high-dose radiation is not used for this population is the size of radiation field which is generally large and usually located closely to intestine or other critical organs. The better survival of patients who could receive SBRT for abdominal lymph node shown in Table 3 could attribute only to the selection bias that the area and volume of the lymph node metastases might be small. Although no definitive reports have described radical radiotherapy for left supraclavicular (“Virchow”) lymph node oligometastases, because it is generally considered that it means a high signal of systemic metastases difficult to survive for the patient. Accordingly, the long survival of the present case in spite of the left supraclavicular lymph node metastases appears to offer important suggestions.

### 3.1. Oligo- but Multisite Metastases: What Is the Rationale for SBRT?

Concerning the relationship between prognosis and primary organ or metastatic site, Milano et al. reported the results of a prospective study with curative-intent SBRT in 121 patients with ≤5 oligometastatic lesions from various primary organs [43]. In the results of that study, patients with primary breast cancer achieved significantly greater local control, progression-free survival, and overall survival rates than those with lung, pancreatic, biliary, or hepatic cancer. They also reported that patients who had adrenal metastases displayed significantly worse prognosis, and patients with lesions confined solely to bone exhibited better survival rates than patients who had other metastatic lesions [23]. Concerning the number of metastases, prognosis is generally regarded as poorer with increasing numbers. However, Milano et al. reported neither the numbers of organs involved nor the numbers of oligometastatic lesions which were significantly associated with measured outcomes, though greater net gross tumor volume (GTV), defined as the sum of GTVs from all treated tumors, was significantly correlated with worse local control [43]. Conversely, Salama et al. reported that the 2-year overall survival rate was better for patients with 1–3 metastases (60.3%) than for patients with 4-5 metastases (21.9%) in a prospective study of SBRT for patients with 1–5 metastatic cancer sites [22].

We do not necessarily recommend aggressive local treatments for patients with repeated oligo-recurrence in multiple organs including adrenal and left supraclavicular lymph node metastases, as in the present case. Actually, poor prognosis was foreseen in the present case because the patient showed four multiple metastases one after another at different sites with short intervals of <1 year. Some investigators have found a disease-free interval of ≥6–12 months as a prognostic factor for improved survival in patients with oligo-recurrent disease [55, 68, 69]. Milano et al. reported an analysis of 32 patients with repeated oligometastases who underwent ≥2 courses of SBRT with curative intent in 121 prospective patients with ≤5 lesions treated using SBRT [60]. In their results, the interval between first and second course of SBRT for new oligometastases was 1–71 months (median, 8 months). The 2-year overall survival and progression-free survival rates for these 32 patients were 65% and 54%, respectively, and patients experienced a trend toward improved overall survival (median, 32 versus 21 months, *P* = 0.13) compared with the other 89 patients who underwent only one SBRT course. The authors concluded that the results have shown that patients fare well with respect to survival and disease control with repeated aggressive SBRT for limited metastases, even after local failure and/or the development of new metastases.

Improvement of systemic chemotherapies, including molecular-targeted therapies, may allow micrometastases to be almost completely absent clinically. Punglia et al. reported that if systemic therapy improves, the role of local therapy would also improve and proposed a figure for this correlation [70]. Rather than eliminating the need for local therapies, improvements in systemic therapies appear to be increasing the prudent utilization of modern local therapies in patients presenting with more advanced cancer [71]. To be sure, in the present case, sequential but systemic oligometastases were fully controlled using radical radiotherapy combined with systemic chemotherapy.

The present patient has been alive and well now without disease. This patient history is beyond our expectation, in a good sense. We attributed the surprising survival from systemic disease in this case to the metastases occurring separately without primary site recurrence (oligo-recurrence state), and cancer cells that were sensitive to not only radiotherapy, but also chemotherapy. Good radio- and chemo-sensitivities were assumed through the response of the left supraclavicular lymph node metastasis to conventional radiotherapy. We also believe the positive and tolerant attitude of the patient might have contributed to the good prognosis in this case.

As the merit of SBRT should be achieved without severe acute or late toxicity, the lower fraction dose in the less hypofractionated schedule, such as in the present case, should be considered for targets near the intestine. In addition, advanced technologies such as volumetric intensity-modulated arc therapy, as well as CT image guidance, will prove highly useful for the purpose of keeping toxicity to a minimum without compromising target dose.

Whether the addition of SBRT can contribute to improved prognosis in patients with repeated metastases remains controversial. The only randomized trials showing improved overall survival with stereotactic irradiation have been in the setting of brain metastases [72]. Ongoing studies are testing the role of SBRT with concurrent systemic therapy in the initial management of patients with limited metastatic NSCLC (NCT00887315) [73].

## 4. Conclusion

A case of a patient with repeated postoperative oligo-recurrence of lung adenosquamous carcinoma to multiple organs who survived long-term following treatment with local radiotherapy and systemic chemotherapy was presented. He developed and was salvaged from multiple metastases one after another at different sites, comprising the adrenal, para-aortic and left supraclavicular lymph nodes, and lung.

Findings in the literature suggest the presence of an oligometastatic state, and local aggressive therapy for oligometastases may improve outcomes, including survival. SBRT has emerged as one option for local therapy against oligometastases in various body sites, most commonly in the lungs and liver. Retrospective studies and clinical trials have demonstrated promising results with the use of SBRT for oligometastases.

However, most reports describing the merits of localized therapies have been based on the results of effects on oligometastases within a single organ. In addition, most studies have relatively included only short follow-up intervals. Longer followup is necessary to better define the role of SBRT in the management of patients with oligometastases. Although further investigation should be undertaken to clarify the benefits, objectives, and methods of SBRT for the treatment of oligometastases, we believe utilization of SBRT would be worthwhile for patients with remote metastases who hope for treatment to acquire better local control and possible longer survival. Even if the disease condition is a little beyond the general definition of oligometastases, as in the present case, SBRT may be beneficial, at least certainly in giving patients courage.

## Figures and Tables

**Table 1:** Studies of stereotactic body radiotherapy for pulmonary oligometastases.

Authors	Study design	Number of patients	Number of metastases in each patient	Primary organ	Dose/fractionation	Followup (months)	Outcomes	Toxicity
Okunieff et al. [20]	Retrospective	42	1 to 5	NA	50 Gy/5 fr (isocenter)	4–61 (median 15)	Crude LC: 94% MS: 23.4 months	Grade 3 (pleural effusion): 1
Kavanagh et al. [36]	Retrospective	34	1 or 2	Lung 15, Colorectal: 9 Others 10	48–60 Gy/4-5 fr (isocenter)	10–80 (median, 27)	2-year LC: 90% 2-year OS: 84.3%	Grade 3 pneumonitis: 3
Nagata et al. [37]	Retrospective	84	1 to 3	Lung 32, Colorectal 11, Kidney 7, others 34	26–48 Gy/1–8 fr (covering PTV)	14–80 (median, 17)	3-year LC: 82% 3-year OS: 16%	NA
Norihisa et al. [38]	Prospective	41	1 or 2	Lung 5, Breast 4, Kidney 4, Others 14	26–37.5 Gy/1–3 fr	2–37 (median, 9)	2-year LC: 80% 2-year OS: 33%	No grade 3, 4
Ernst-Stecken et al. [42]	Prospective	61	1 or 2	Lung 31, Colorectal 8, Others 22	12–30 Gy/fr (isocenter)	2–82 (median, 14)	2-year LC: 74% 2-year OS: 65%	Grade 3 pneumonitis: 3%
Hof et al. [44]	Prospective	38	1 to 3	Colorectal 9, Sarcoma 7, Kidney 7, others 15	48–60 Gy/3 fr (covering PTV)	6–48 (median, 16)	2-year LC: 96% MS: 19 months	Grade 3 pneumonitis: 4 Grade 3 chest wall: 2

Abbreviations: NA: not available; LC: local control rate; OS: overall survival rate; LPFS: locally progression-free survival rate.

**Table 2:** Reports of stereotactic body radiotherapy for adrenal oligometastases.

Authors	Study design	Number of patients	Primary organ	Dose/fractionation	Followup (months)	Outcomes	Toxicity
Nuyttens et al. [54]	Retrospective	30	Lung 20, others 10	16–50 Gy/4–10 fr (isocenter)	1–35	1-year LC: 44% 1-year OS: 55%	No grade > or =2
Tanvetyanon et al. [55]	Retrospective	48	Lung 24, Colorectal 12, others 12	36 Gy/3 fr (covering PTV)	3–63 (median, 17)	2-year LC: 90%	Adrenal deficiency: 1
Chawla et al. [56]	Retrospective	7	Lung 19	30–60 Gy/1–27 fr	NA	2-year OS: 33%	NA
Casamassima et al. [57]	Retrospective	19	Lung 4, others 3	16–27 Gy/1–3 fr (covering PTV)	1–60 (median, 38)	1-year LC: 63% MS: 8 months	NA

Abbreviations: LC: local control rate; OS: overall survival rate; NA: not available; MS: median survival time.

**Table 3:** Reports of stereotactic body radiotherapy for isolated abdominal lymph node metastases.

Authors	Study design	Number of patients	Primary organ	Dose/fractionation	Followup (months)	Outcomes	Toxicity
McCammon et al. [48]	Retrospective	30	Uterine cervix: 28 Uterine corpus: 2	30–45 Gy/3 fr (covering PTV) (+EBRT 27–45 Gy)	2–65 (median; 16)	4-year OS: 50.1% 4-year LC: 67.4	No severe complication on intestine
Onishi et al. [49]	Retrospective	7	Stomach: 7	45–51 Gy/3 fr (covering PTV)	19–33 (median: 26)	CR: 5/7, PR: 2/7 3-year OS, PFS: 43%, 29%	No severe complication
Choi et al. [50]	Retrospective	7	Colorectal: 7	36–51 Gy/3 fr (covering PTV)	15–70 (median; 26)	1-, 3-year OS: 100%, 71.4% MS: 37 months,	Grade 4 intestine: 1

Abbreviations: LC: local control rate; MS: median survival time; OS: overall survival rate; CR: complete remission; PR: partial remission; PFS: progression free survival.
